# The Surgical Diseases of the Genito-Urinary Organs, Including Syphilis

**Published:** 1888-07

**Authors:** 


The Surgical Diseases of the Genito-Urinary Organs, Including Syphilis. By E. L. Keyes, A. M., M. D. New York: D. Appleton & Co.

When Van Buren and Keyes published their great work on genito-urinary diseases in 1874, it seemed as if nothing further could be desired in this class of diseases. It at once won for itself a high place in medical literature. But during the past fourteen years, numerous and important advances have been made in some of the diseases treated of in this work; for instance, lithotrity has given way to litholapaxy, supra-pubic cystotomy has again come into favor, and Neisser's and Lustgarten's discoveries have given great impetus to the study of the etiology of gonorrhœa and syphilis.

The profession will therefore gladly welcome the revised edition of this profound work. As the hand of the great Van Buren is still forever, the task has been accomplished by the gifted Keyes, and in a way that proves him a worthy successor to his "dear old master." This revision has really involved a re-writing of the whole book. The work is now thoroughly abreast of the times, and will continue to occupy its old place as one of the very best books on genito-urinary diseases in the English language.

